# Evaluation of a Wi-Fi Signal Based System for Freeway Traffic States Monitoring: An Exploratory Field Test

**DOI:** 10.3390/s19020409

**Published:** 2019-01-20

**Authors:** Fan Ding, Xiaoxuan Chen, Shanglu He, Guangming Shou, Zhen Zhang, Yang Zhou

**Affiliations:** 1 Department of Civil and Environmental Engineering, University of Wisconsin, Madison, WI 53706, USA; zhen.zhang@wisc.edu (Z.Z.); zhou295@wisc.edu (Y.Z.); 2 Ford Motor Company, Dearborn, MI 48126, USA; xchen172@ford.com; 3 School of Automation, Nanjing University of Science and Technology, Nanjing 210000, China; slhemickey@126.com; 4 School of Cyberspace Security, Southeast University, Nanjing 210000, China; 15151853118@163.com

**Keywords:** Internet of Things (IoT), Wi-Fi signal detector, traffic characteristics monitoring, speed detection, performance evaluation

## Abstract

Monitoring traffic states from the road is arousing increasing concern from traffic management authorities. To complete the picture of real-time traffic states, novel data sources have been introduced and studied in the transportation community for decades. This paper explores a supplementary and novel data source, Wi-Fi signal data, to extract traffic information through a well-designed system. An IoT (Internet of Things)-based Wi-Fi signal detector consisting of a solar power module, high capacity module, and IoT functioning module was constructed to collect Wi-Fi signal data. On this basis, a filtration and mining algorithm was developed to extract traffic state information (i.e., travel time, traffic volume, and speed). In addition, to evaluate the performance of the proposed system, a practical field test was conducted through the use of the system to monitor traffic states of a major corridor in China. The comparison results with loop data indicated that traffic speed obtained from the system was consistent with that collected from loop detectors. The mean absolute percentage error reached 3.55% in the best case. Furthermore, the preliminary analysis proved the existence of the highly correlated relationship between volumes obtained from the system and from loop detectors. The evaluation confirmed the feasibility of applying Wi-Fi signal data to acquisition of traffic information, indicating that Wi-Fi signal data could be used as a supplementary data source for monitoring real-time traffic states.

## 1. Introduction

Traffic state monitoring is accepted as an extremely important topic, especially when the whole transportation community is studying automated travel and finding solutions for smart cities. Developed and conventional devices such as loop detectors [1], microwave radars [2], and cameras [3,4] have been widely deployed. Monitoring functions and accuracy of these devices are mainly subject to the direct impacts of individual equipment. Furthermore, installation of these devices requires coordination with roadside power lines, increasing the costs of installation and maintenance [5]. In recent decades, data sources such as GPS data [5,6,7], cellular data [8,9,10,11], Bluetooth data [12,13,14,15,16], and Wi-Fi signal data [17,18,19] have gained attention from the community for traffic state monitoring. Conventionally, traffic characteristics are directly collected from roadside equipment. Nowadays, researchers are devoted to implementing mining algorithms to extract traffic state information from novel data sources, in which normally unique identifications, timestamps, and potential locations are provided.

Positioning methods of different data are obtained from a variety of probe techniques, among which accurate positioning information is generally considered to be obtained from GPS data. However, to acquire GPS data, applications on either smart phones [6] or GPS embedded devices on vehicles [20,21] are needed. As a result, GPS data are normally collected from a specific, small set of the whole traffic population, limiting coverage and introducing potential systematic bias. In the last two decades, another attractive data source, cellular data, has been applied for estimation of traffic states [8,9]. Cellular data, as an extremely big dataset, is able to significantly expand monitoring coverage at comparatively low cost, especially for large-scale freeway networks. Since location information of cell phones in the communication network is not directly provided, handover probes are proposed for determining potential phone locations, which, however, are not precise and hence may cause estimation errors [9]. In the meantime, both GPS and cellular techniques for locating individual cellphones arouse privacy concerns, further increasing the cost and difficulty of data collection.

## 2. Related Works

With the development of wireless technologies, new wireless sensors are being introduced and widely studied in the transportation community for traffic state monitoring [22,23]. New wireless sensors provide opportunities for researchers and practitioners to comprehensively perceive the whole transportation network, and to collect diverse information. Bao et al. [24] presented a solution to accurate counting of vehicles under jam flow conditions through wireless magnetic sensors. To identify vehicles’ locations and corresponding traffic measurements, Jeon et al. [25] employed wireless ultrasonic sensors to laterally scan multiple lanes. Due to luxury vehicles equipped with Bluetooth modules for communication purposes, some researchers have installed roadside Bluetooth sensors and utilized Bluetooth fingerprints to collecte real time freeway travel time [12], to calculate the origin–destination matrix [13], and to estimate traffic speed [14]. A study proved no statistically significant difference between the performance of applying GPS data and Bluetooth data when observing highway travel time [15]. However, the sampling rate of Bluetooth data was found to be within the relatively low range of 2.0–3.4%, in terms of the main traffic stream [12]. Furthermore, it was reported that, because of the specific protocol, Bluetooth traffic sensors are subject to limitations such as default interference, small detection range, and high data-transferring latency [26].

With the trend of ‘Internet going mobile’, Wi-Fi signal data, as smart phone fingerprints, have become an attractive data set for studies on the dramatically increases and strong relevance between human activities and phones nowadays. Abedi et al. [17] estimated travel time of pedestrians and cyclists through Bluetooth and Wi-Fi media access control (MAC) addresses. Ryeng et al. [18] evaluated the performance of a bicycle speed collection system based on Bluetooth and Wi-Fi sensor data. Danalet et al. [27] proposed a Bayesian approach to obtain pedestrian activity episodes from Wi-Fi signatures. Some studies have also conducted accurate indoor localization using characteristics and properties of Wi-Fi signals [28,29,30]. In addition, Shiravi et al. [19] creatively performed a field test to estimate intersection travel time in a residential area, using combined data of Bluetooth and Wi-Fi. Through the findings, they pointed out the significant differences in market penetration rates between Bluetooth and Wi-Fi, as well as higher sample sizes of Wi-Fi data than of Bluetooth data. In terms of monitoring traffic speeds through novel data, Bar-Gera [8] evaluated the performance of a cellular phone-based system in measuring traffic speeds in Israel. The average relative absolute differences found ranged from 6% to over 20% compared to loop data. Herrera et al. [6] evaluated the performance of a GPS-enabled mobile phone based traffic speeds monitoring system. In that study, compared to loop data, the absolute speed differences of 70% of observations were less than 5 mph under the traffic speeds of over 55 mph. Haghani et al. [12] also assessed traffic speeds collected from Bluetooth sensors. When traffic speeds were above 60 mph, the average absolute speed error reached 4.8 mph, compared to corresponding loop detector data.

In terms of collecting traffic characteristics from Wi-Fi data, more works are still needed, so as to evaluate the performance of a Wi-Fi signal-based traffic monitoring system; to quantitatively compare the volumes of Wi-Fi signal and traffic to evaluate the proportion of actual vehicles detected by a Wi-Fi signal-based system; and to find out an improved algorithm instead of a basic Wi-Fi signal scanning algorithm, for efficient and productive collection of Wi-Fi signal data. This paper introduces a newly designed IoT and green technology-based Wi-Fi signal detector, on the basis of which a traffic monitoring system was implemented. To the best of our knowledge, it is the first practical field test on evaluating a Wi-Fi signal-based method that is used for monitoring traffic speeds on freeways.

The remaining paper is organized as follows: Section 3 presents the main scheme of the proposed Wi-Fi signal-based system. Section 4 introduces the design of an IoT green technology-based Wi-Fi signal detector. The details of the Wi-Fi raw data filtering method and the traffic speed mining algorithm are provided in Section 5. In Section 6, based on a field test conducted in China, the performance is evaluated, and the penetration rate of vehicles detected by Wi-Fi signal sensors is discussed. Finally, the main conclusions and potential future research directions are given in Section 7.

## 3. Scheme of the Wi-Fi Signal Based Traffic Monitoring System

The proposed Wi-Fi signal-based traffic monitoring system is a passive platform for data collection and processing. To obtain accurate results and good performance, four steps are taken to deal with the four principal tasks, including data collection, data cleaning, data mining, and traffic state extraction. Several IoT-based and solar energy supported Wi-Fi signal detectors were designed, assembled, and then installed on the roadside to collect Wi-Fi signals. Due to errors and interferences in raw data, a filtering algorithm was proposed for elimination of incorrectly formatted records, types of duplicates, and invalid and unpaired records during future calculations. After filtering, Wi-Fi signal data still failed to directly provide traffic information. Thus, a mining algorithm was implemented to build the relationship between Wi-Fi signals and traffic. Traffic state data that can be extracted from Wi-Fi signal data include average link speeds, traffic volumes, travel time, and movement trajectories. Researchers have studied on topics such as revealing travel time from Wi-Fi signal data [17,19]. This paper mainly focuses on extraction of traffic speeds and discussion of the possibility of using Wi-Fi volumes to estimate traffic volumes. The details will be given in the following sections.

## 4. Design of the Wi-Fi Signal Detector

**Figure 1:**
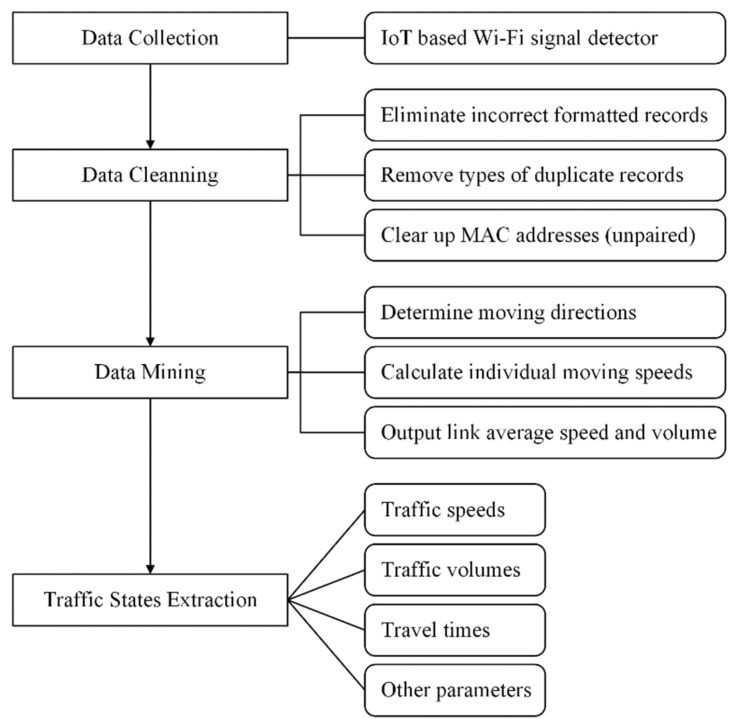
The scheme diagram of the proposed Wi-Fi signal-based traffic monitoring system.

The basic unit for a Wi-Fi signal-based traffic monitoring system is the signal detector. With the recent improvement of IoT technologies, a Wi-Fi signal detector was designed and developed in the current study. It was powered by solar energy, and transmitted data through a wireless network (Figure 1), which helped easily and flexibly install such detectors at low cost. Meanwhile, with correct deployment strategies, a wireless sensor network (WSN) with Wi-Fi signal detectors as sensing nodes could comprehensively acquire multiple types of traffic details, including average link speed, average travel time, traffic volume, and individual route trajectories.

**Figure 2:**
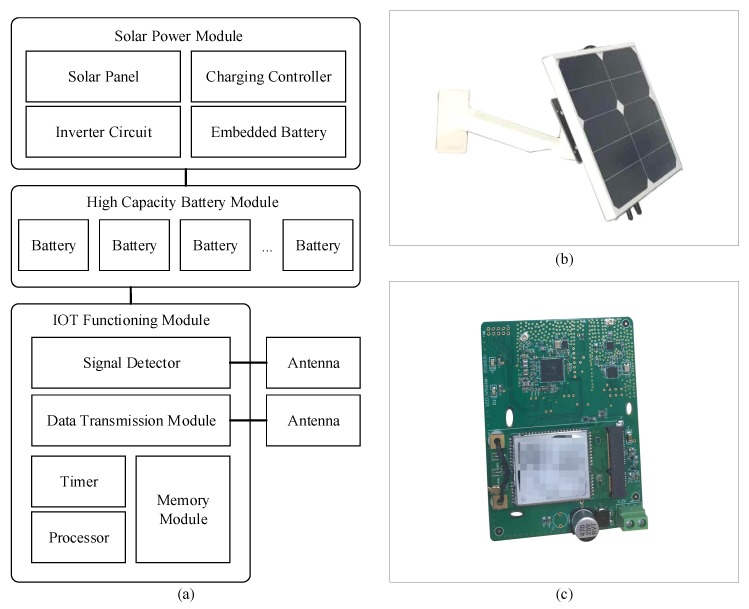
(**a**) The abstract architecture of the designed detector, (**b**) the appearance of the detector, and (**c**) the principle circuit for the detector.

The Wi-Fi signal detector consists of three main modules, which are a solar power module, a high capacity battery module, and an IoT functioning module (Figure 2a).

• Solar power module

To realize sustainability and quick installment, solar energy was used to power the designed Wi-Fi signal detector. This avoided the use of roadside electricity power networks, which could be costly for developing countries. The solar power module contains four major components, a panel, a charging controller, an inverter circuit, and a battery. It is a mature and widely used solution; therefore, details are omitted here.

• High capacity battery module

Emerging battery technology has already achieved high energy density. Unlike conventional lead–acid batteries, high capacity batteries are able to store large amount of energy within a small volume. At present, a fully charged battery module could support the Wi-Fi signal detector to operate for nearly one week, thus minimizing power consumption.

• IoT functioning module

The IoT (Internet of Things) functioning module is the core of the designed detector. In this module, the signal detector scans corresponding Wi-Fi frequency channels and records detected MAC addresses; the processor marks each MAC address with the detector number and a received timestamp based on the timer; finally, the data transmission module sends records to the backend database. Table 1 provides the list of collected attributes for the signals.

**Figure 3:**
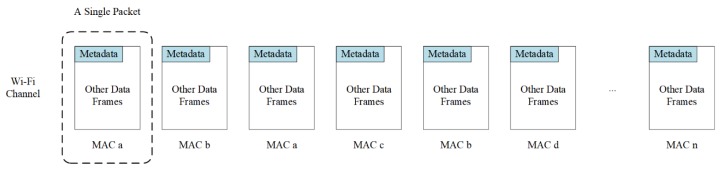
Illustration of an example state for data packets in a Wi-Fi channel.

To collect Wi-Fi signal data, it is necessary to scan corresponding Wi-Fi channels. Figure 3 illustrates an example state for one Wi-Fi channel. For the Wi-Fi protocol, a single data packet of about 1400 bytes stores main data information and other protocol interface information. However, from the perspective of monitoring traffic states, most information of the packet, such as private data payload, is redundant, other than the MAC addresses. The scanning method was optimized to monitor wireless network traffics for improvement of scanning effectiveness and collecting efficiency. Only a few bytes of metadata containing MAC address for each packet are inspected by the new process. The signal probe detector was set to silent mode, thereby leading to significant power consumption reduction. The optimized scanning solution avoided unnecessary data scanning and storage, improving the scanning utility and efficiency, and reduced miss scanning rate. The data collected were transmitted through the communication network (4G network), during which the timer of a detector was synchronized based on the network time protocol. Moreover, since the communication network was working on a different frequency band compared to Wi-Fi signals, the signal acquisition antenna would not be interfered with by the data transmission antenna.

Furthermore, in Wi-Fi active devices, there are two major operating statuses, the searching status and the connecting status. Under the connecting status, devices transmit data through only one channel; by contrast, devices send out searching messages (frames) through all major channels under the searching case. Considering the environment of freeways, Wi-Fi active devices on the freeway should mainly belong to the searching mode. Practically, rather than monitoring all channels, focusing on one major channel ensures the sampling rate and achieves high sample size.

## 5. Proposed Filtering and Mining Algorithm

### 5.1. Filtering Raw Wi-Fi Signal Data

In addition to illustrating an example state of Wi-Fi channel, Figure 3 also presents the fact that packets belonging to different devices are randomly arranged in a channel, since devices are equally served to share channel resources [31]. In the meantime, since in the scanning process only detected MAC addresses were collected, but duplicates were not removed, a filtering method was proposed to pre-process raw data, thus removing not only MAC addresses with a single record but also incorrect formatted and duplicate records.

• Error records

In communication systems, faults may occur occasionally, leading to incorrect formatting of records such as irregular MAC addresses, unreasonable timestamps, and nonsexist detector numbers. These error records are easily identified and removed from the raw data set by simple checking rules.

• Duplicate records

As abovementioned, the optimized scanning method realized improvements in scanning effectiveness and collecting efficiency. However, this improvement also increased the number of duplicate records sent by each device in the Wi-Fi channel. Thus, arbitrary rules were implemented to de-duplicate. Only one of records with identical MAC addresses, timestamps, and detector numbers was reserved, and others were directly eliminated. For records with identical MAC addresses and detector numbers, if their timestamps were within a short time window, the first record (minimal timestamp) was kept. To determine the length of such a short time window, speed limitation and detector coverage were taken into account. For records with identical MAC addresses and timestamps but different detector numbers (accumulative mileage), those with smaller detector numbers (smaller accumulative mileage) were kept. Detector numbers (accumulative mileages) were assigned (calculated) based on one moving direction, since detectors were linearly installed on the segment.

• MAC addresses with a single record

As indicated in previous studies [17,19], to determine the moving directions of Wi-Fi embedded devices, at least two signal detectors are required, regarding the detection and pairing mechanism. The pairing of signal data depends on unique identification information (MAC address). Therefore, MAC addresses with a single record (after de-duplicate) were not useful, and were therefore excluded from the raw data set.

### 5.2. Extracting Traffic Details

After filtering, Wi-Fi signal data still failed to directly and intuitively present any traffic information unlike loop data and GPS data. To extract objective traffic state information, such as average link speed, a mining algorithm (Algorithm 1) was proposed as follows.
**Algorithm 1.****Input:**  $r_{t}$ (all records of interval *t*) and $r_{t-1}$ (all records of interval *t*−1)**Output:**  $vs_{t}$ and N for each direction over interval *t*
  **FOR** each MAC identification *mac* in $r_{t}$
    $r_{mac,t}\;=\;\{\forall \;record\;|\;record\in mac\cap record\;\in r_{t}\}$
    $r_{mac,t-1}\;=\{\forall \;record\;|\;record\in mac\cap record\;\in r_{t-1}\}$
    Sort $r_{mac,t}$, $r_{mac,t-1}$ by ts in ascending order
    $∆d_{mac,t}=d_{last\left(r_{mac,t}\right)}-d_{first\left(r_{mac,t}\right)}$
    $∆ts_{mac,t}=ts_{last\left(r_{mac,t}\right)}-ts_{first\left(r_{mac,t}\right)}$
    **IF**
$∆d_{mac,t}=0$
        $∆d_{mac,t}=d_{last\left(r_{mac,t}\right)}-d_{first\left(r_{mac,t-1}\right)}$
        $∆ts_{mac,t}=ts_{last\left(r_{mac,t}\right)}-ts_{first\left(r_{mac,t-1}\right)}$
    **END IF**
    **IF**
$∆d_{mac,t}>0$
        $vs_{mac,t}=∆d_{mac,t}\;/∆ts_{mac,t}$
        Assign $vs_{mac,t}$ to *positive direction set*
    **ELSE IF**
$∆d_{mac,t}<0$
        $vs_{mac,t}=-∆d_{mac,t}\;/∆ts_{mac,t}$
        Assign $vs_{mac,t}$ to *negative direction set*
    **ELSE**
        Filter *mac*
    **END IF**
  **END FOR**
  **FOR** each direction set      $vs_{t}=\frac{1}{N}\overset{N}{\underset{mac}{\sum }}vs_{mac,t}+N\cdot \sigma^{2}/\overset{N}{\underset{mac}{\sum }}vs_{mac,t}$
  **END FOR**

Here, the basic merge sorting algorithm was used to order the input data. The functions first(·) and last(·) were used to extract the first and last record from a record set, respectively. Each detector was assigned an accumulative mileage, and the mileage for the corresponding detector of a single record is represented by $d_{record}$. The distance between the detector of the last record, $d_{last\left(re_{mac,t}\right)}$, and the first record, $d_{first\left(re_{mac,t}\right)}$, is represented by $∆d_{mac,t}$ for MAC identification *mac* during interval *t*. Time difference is referred to by $∆ts_{mac,t}$. If $∆d_{mac,t}$ equals zero, the record set of the previous interval, *t*−1, is retrieved for MAC identification *mac*, and both the time difference and the distance correspondingly are re-calculated. The speed of *p* during interval *t*, represented by $vs_{mac,t}$, is then calculated and assigned to one direction set. The total number of valid moving devices (valid Wi-Fi signal volumes) for each direction, denoted as *N*. σ, is the standard deviation for $vs_{t}$.

Since the actual location cannot be accurately estimated, the mining algorithm utilizes sorted paired records of a MAC address to determine the moving direction and to compute moving speed, based on the corresponding detector location of records. Though some studies have applied signal strength to accurate localization [32,33], such processes are mainly designed for indoor cases. In this study, the corresponding location of detectors for each record was used to approximately represent the MAC device location, which may intuitively introduce errors. Thus, a field test was conducted to evaluate the performance of the proposed mining algorithm.

## 6. Field Test

### 6.1. Testbed and Data Collection

The testbed of this research was a 1 km long, bi-directional, four-lane segment of G2 Jinghu Expressway, close to Yong’an River Bridge in Jiangsu, China. The lane width is 3.75 m and the shoulder is 2 m. A total of six IoT-based detectors were uniformly installed on the northbound roadside guardrails. This installation required detectors not only to cover the link and calculate speed, but also to enlarge the number of Wi-Fi signal samples, especially when Wi-Fi signals were not always captured absolutely under high speed moving scenarios (over 60 km/h). In the meantime, considering the covering radius of a single IoT-based detector is 60 m, each detector was able to realize monitoring of both directions. Such a coverage radius would not lead to overlapping zones for adjacent detectors.

**Figure 4:**
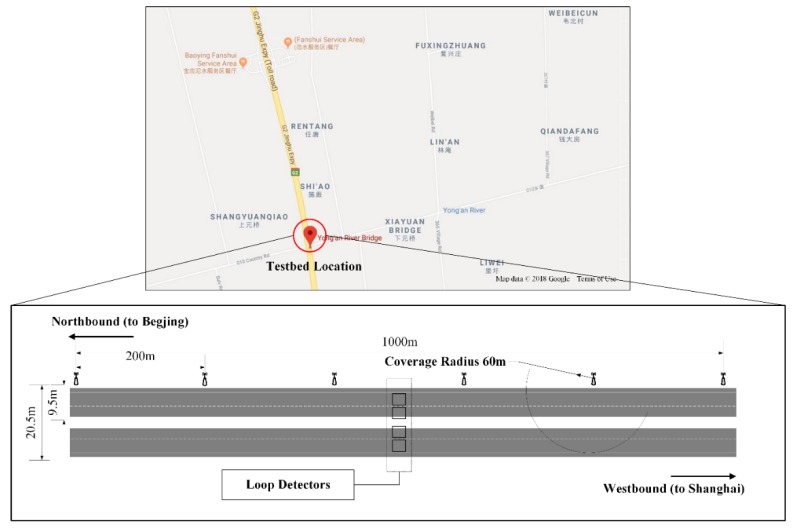
General view of the research corridor as well as the detailed layout and indications of the testbed (*Note: background map photo is from Google Maps*).

A group of loop detectors were installed in the middle of the selected link. The loop detector data were considered as ground truth data for the performance validation. Figure 4 presents the general view and layout of the testbed. Data from all devices, including both loop detectors and IoT-based detectors, were collected from 00:00 April 22 to 23:59 April 23 in 2017. After eliminating outliers for all types of raw data, the data were aggregated for each 5 min time interval. Studies have proven the feasibility and practicality of an update window of 5 min for guaranteeing the effectiveness of traveler information in transportation networks [34,35].

### 6.2. Results

**Figure 5:**
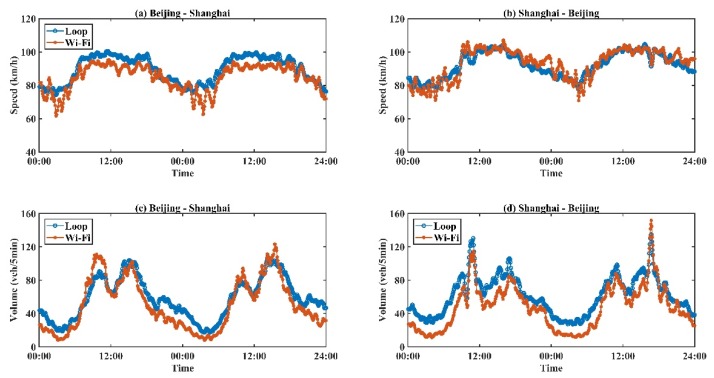
Comparisons between speed and volume obtained from Wi-Fi signal-based system and loop detectors. (**a**) Comparison results between speed obtained from loop detectors and Wi-Fi signals for Beijing to Shanghai over time, (**b**) Comparison results between speed obtained from loop detectors and Wi-Fi signals for Shanghai to Beijing over time, (**c**) Comparison results between volumes obtained from loop detectors and Wi-Fi signals for Beijing to Shanghai over time, (**d**) Comparison results between volumes obtained from loop detectors and Wi-Fi signals for Shanghai to Beijing over time.

To further de-noise the data and reduce the impacts of outliers, a seasonal trend decomposition based on the loess (STL) decomposition procedure [36] was performed on all state data for both outputs of the proposed system, and aggregated loop data over two directions. Figure 5 shows the comparisons of speed and volume between the results of Wi-Fi signal and aggregated loop. The proposed system demonstrated good performance for distinguishing moving phones of different directions, and computing traffic states based on Wi-Fi signal data.

Corresponding results of two data sources were found to be highly correlated (Figure 5). Specifically, traffic speed results obtained from the Wi-Fi signal-based system were almost equal to those from loop detectors. Moreover, the volumes of Wi-Fi signals and vehicle counts were generally consistent and identical. However, the differences became obvious and significant, especially during the night period. Detailed accuracy analysis and error sources are provided in the next section.

### 6.3. Accuracy Analysis

**Figure 6:**
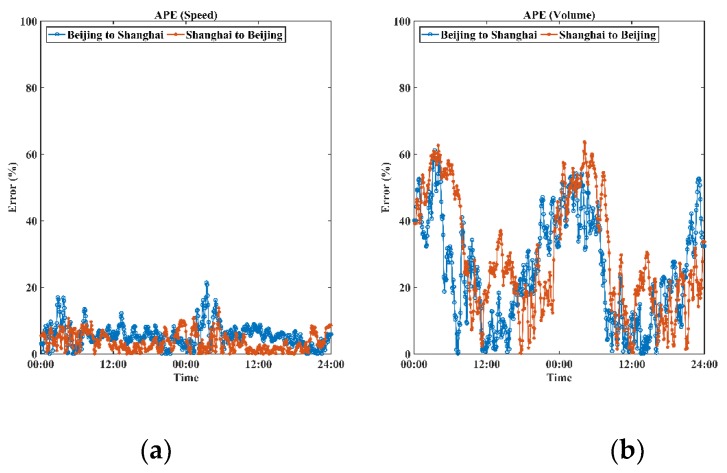
Details of absolute percentage errors over time for speed (**a**) and volume (**b**) between loop data and outputs of the Wi-Fi signal-based system.

To evaluate the global performance, the mean absolute error (MAE), the mean square error (MSE), and the mean absolute percentage error (MAPE) were selected as accuracy evaluation indicators. The absolute percentage error (APE) was also employed to provide detailed difference analyses for both speed and volumes of the two sources over time, as shown in Figure 6. Corresponding equations for the indicators are expressed as follows.

(1) $$\mathrm{MAE}=\frac{1}{n}\overset{n}{\underset{t=1}{\sum }}\left|\hat{v_{t}}-v_{t}\right|$$

(2) $$\mathrm{MSE}=\frac{1}{n}\overset{n}{\underset{t=1}{\sum }}{\left(\hat{v_{t}}-v_{t}\right)}^{2}$$

(3) $$\mathrm{MAPE}=\frac{1}{n}\overset{n}{\underset{t=1}{\sum }}\frac{\left|\hat{v_{t}}-v_{t}\right|}{\hat{v_{t}}}$$

where $\hat{v_{t}}$ and $v_{t}$ are the *t-th* observed values collected from loop detectors and from the Wi-Fi signal-based system for interval *t*, respectively.

Table 2 and Table 3 present the global performance of speed and volume for both directions. The performance of speed estimation based on Wi-Fi signal data was found to be reliable and acceptable. For both directions, the mean absolute errors were lower than 5 km/h, indicating insignificant differences between two sources. This might be because the speed obtained from the loop detectors originally contained certain errors. On the other hand, volume differences were relatively significant, since volumes of the Wi-Fi signal-based system were obtained by counting moving electronic devices rather than moving vehicles, compared to loop data. To estimate vehicle volumes from outputs of the Wi-Fi signal-based system, further modeling methods should be proposed. This paper focuses on evaluating the performance of the Wi-Fi signal-based system instead of proposing a traffic volume estimation method; discussion of the modeling part was therefore omitted.

**Figure 7:**
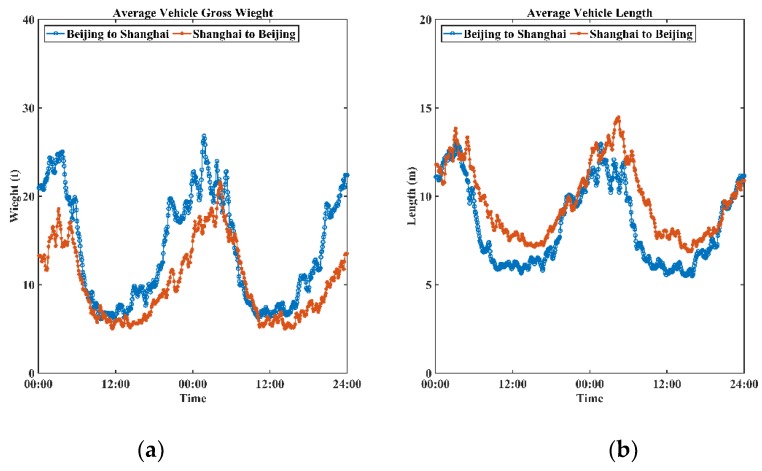
Demonstrations of average vehicle gross weight (**a**) and length (**b**) over time.

Specifically, the differences between the two sources may be potentially explained by, but not limited to, the following reasons:

- In this study, data collected from loop detectors were employed as ground truth data for validating the performance of the Wi-Fi signal-based system. Although such loop detectors were newly installed, loop data obtained may still contain noise and interferences due to communication errors and detector malfunctions [37];
- Empirically, heavy vehicles dominate night-time traffic. This phenomenon also exists on the G2 Jinghu Expressway (Figure 7). The average length and gross weight of vehicles (data obtained from loop detectors and calculated through arithmetic average method) both increased significantly during the night period. As the Wi-Fi signal detectors were installed on roadside guardrails, heavy vehicles, particularly cargo containers, could easily block signals, thereby reducing the number of captured Wi-Fi signals;
- The Wi-Fi signal data failed to provide exact location information of signal sources due to the passive sensing process. The proposed algorithm substituted the positioning information of records by the detector location. This substitution indeed introduced certain systematic errors, though the coverage of each detector was comparatively small, especially when vehicles passed the area at high speed (over 60 km/h);
- Under high speed scenarios (over 60 km/h), Wi-Fi signals were not 100% captured, even though the scanning mechanism of IoT detectors was optimized to reduce the reaction time. This fact may lead to total counts of unique Wi-Fi devices less than volumes of vehicles;
- Nowadays, in a single vehicle, there might be multiple Wi-Fi embedded electronic devices sending out various signals. In the meantime, it is almost impossible to build direct mapping relationships between Wi-Fi signals and vehicles. These reasons would result in a number of detected on-route electronic devices higher than that of vehicles;
- Certainly, there also might be no electronic devices in a vehicle, especially when only a driver was in the vehicle. This phenomenon would cause the volume of Wi-Fi signals to be less than that of vehicles.

### 6.4. Discussion of the Penetration Rate of Vehicles Being Detected

**Figure 8:**
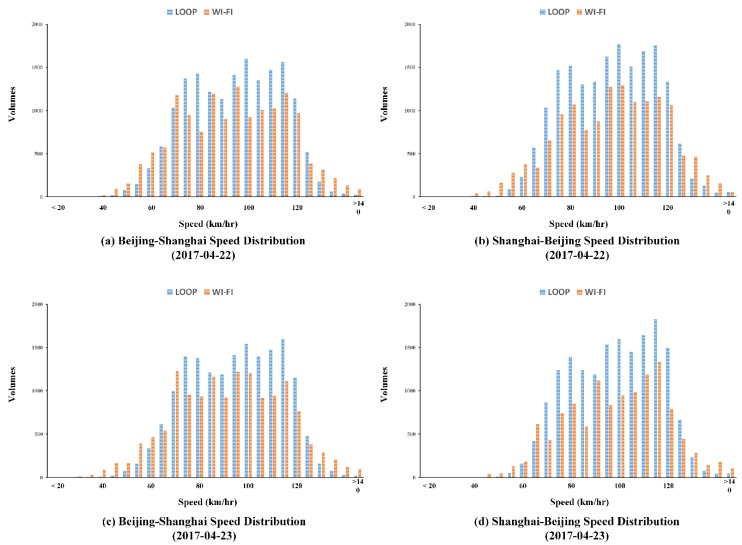
Comparison of speed distributions between the numbers of vehicles and unique MAC addresses (after filtering and mining). (**a**) Speed distribution comparison between loop detector results and Wi-Fi signal based results for Beijing to Shanghai over 22 April 2017, (**b**) Speed distribution comparison between loop detector results and Wi-Fi signal based results for Shanghai to Beijing over 22 April 2017, (**c**) Speed distribution comparison between loop detector results and Wi-Fi signal based results for Beijing to Shanghai over 23 April 2017, (**d**) Speed distribution comparison between loop detector results and Wi-Fi signal based results for Shanghai to Beijing over 23 April 2017.

As mentioned in the discussion of potential error sources, intuitively, Wi-Fi signal data may involve significant noise, considering the existence of one-to-many relationships between vehicles, especially coach buses, and Wi-Fi embedded devices. The proposed filtering and the mining algorithms were employed to eliminate such errors and interferences. However, it is still necessary to discuss the relationships between vehicle volumes and the number of unique MAC addresses. To reveal such relationships, speed distributions of two resources for each day during the research period were examined. Figure 8 shows the comparison of speed distributions between volumes of vehicles and unique MAC addresses. As also indicated by Figure 5 and Table 3, obvious differences between two volumes were observed. Overall, volumes of Wi-Fi signals were less than vehicle volumes. The distribution of vehicle speeds obtained from loop detectors was globally concentrated with higher peaks, while that of unique MAC addresses was comparatively gentle. These differences could be explained by different detection mechanisms: loop detectors collected vehicles’ instantaneous spot speeds; the proposed system calculated average speeds of individual unique addresses.

To further evaluate the detection rate of IoT-based detectors, the total distinct MAC addresses collected by each detector for both directions were computed. Table 4 presents the number of total distinct detected identifications for each IoT-based detector over two directions, respectively. The result that the number of total detected unique addresses of Shanghai to Beijing was greater than that in the other direction is consistent with the result of vehicle observations from loop data. Differences of total detected distinct MAC addresses among IoT detectors were not significant for both directions. Though an individual IoT detector only detected small number of distinct MAC addresses comparing to loop data, a group of multiple IoT detectors could reach a high detection rate (Figure 8). Therefore, the detection rate of IoT detectors for individual Wi-Fi embedded electronical devices was considered stable and reliable under high speed moving scenarios (over 60 km/h).

### 6.5. Specifications of the Proposed System and the Wi-Fi Signal Detector

Based on results obtained from the field test, the specifications of the proposed traffic monitoring system were summed up. The maximum speed detected was 144 km/h. The minimum was 3.4 km/h due to a pedestrian walking along the shoulder (often occurring in rural areas). Furthermore, theoretically, the shortest time difference between records was 1 s and the distance between adjacent detectors was 200 m, thus the maximum detected speed could reach 200 m/s. This is far larger than vehicle capability, indicating that the system could cover extreme speed cases. In addition, based on the mining algorithm, the largest time difference was 10 min when applying the extended detection window; thus, the minimum detected speed of the system is 20 m/min in theory. As aforementioned, some specifications for IoT-based Wi-Fi signal detectors are listed in Table 5.

## 7. Conclusions and Future Work

This paper introduces a Wi-Fi signal-based system to monitor traffic states of freeway segments. An IoT-based and solar energy supported detector was designed to collect corresponding Wi-Fi signal data. This compact detector was characterized by low power consumption, easy and flexible deployment, and reduced cost of installation and maintenance. In addition, a filtering method was proposed to clean raw Wi-Fi signal data and a mining algorithm was used to extract traffic characteristics. A practical field test was also performed on a busy freeway, G2 Jinghu Expressway in Jiangsu province, China. Through the field test results, the proposed system could reach 3.18 km/h for MAE at best in terms of traffic speed, compared to data obtained from loop detectors. This evaluation indicates that the Wi-Fi signal-based system could provide promising and accurate traffic information.

The evaluation in this research proved the feasibility of applying Wi-Fi signal data for traffic information acquisition. Future studies may focus on the following directions.

(1) The performance of new designed detectors may vary because of environmental factors. Therefore, it is necessary to further evaluate the performance of the Wi-Fi signal-based system over a long time period, covering different weather conditions and seasons.
(2) For future field tests, it is suggested to prioritize acquisition of a precise detection rate of the detector under different speed scenarios. In addition, efforts should be made to examine the relationships between vehicles and mobile devices.
(3) Traffic characteristics are highly correlated to road types and geometric design. The current work only covers freeway segments. To evaluate the system for ramps and urban expressway will be essential for proving the feasibility of the proposed system in other complex and congested conditions.
(4) Elegant algorithms, such as deep learning-based methods, have been proposed to improve the quality and accuracy of estimated traffic states. Investigations of application of these complex algorithms to Wi-Fi signal data will help to further improve the performance of the system, and especially to estimate traffic volumes from Wi-Fi signal volumes.

## Figures and Tables

**Table 1:** List of collected Wi-Fi signal data attributes.

Attribute Description	Example
MAC address	35:69:15:9c:7c:8a
Timestamp	2017-04-22 11:12:13
Detector Number	320101
Signal Strength (dB)	60

**Table 2:** Global speed error analysis for both directions.

	MAE	MSE	MAPE
Beijing–Shanghai	4.930	32.343	5.52
Shanghai–Beijing	3.180	15.468	3.55

**Table 3:** Global volume error analysis for both directions.

	MAE	MSE	MAPE
Beijing–Shanghai	11.634	175.685	24.68
Shanghai–Beijing	15.155	274.149	29.24

**Table 4:** Number of total distinct detected identifications for Internet of Things (IoT) detectors during the field test.

Detector No.	1	2	3	4	5	6	Loop
Beijing–Shanghai	13,395	13,248	13,106	12,421	12,892	13,601	32,394
Shanghai–Beijing	14,252	15,049	14,621	14,536	14,466	14,365	35,660

**Table 5:** Specification table of the proposed system and IoT-based Wi-Fi signal detector.

**System Features**	
Reported Time Window	5 min
Minimum Detected Speed	144 km/h
Maximum Detected Speed	3.4 km/h
Minimum Detected Speed (in theory)	20 m/min
Maximum Detected Speed (in theory)	200 m/s
**Wi-Fi Signal Detector Features**	
Power Consumption	≤0.4 W
Initial Battery Capacity	≥3350 mAh
